# The Feeling of “Face” in Confucian Society: From a Perspective of Psychosocial Equilibrium

**DOI:** 10.3389/fpsyg.2016.01055

**Published:** 2016-07-19

**Authors:** Kuei-Hsiang Han

**Affiliations:** Graduate Institute of Educational Psychology and Counseling, Tamkang University, New Taipei CityTaiwan

**Keywords:** having face, losing face, modesty, psychosocial equilibrium, self-enhancing

## Abstract

Previous research on the feeling of “face” has long described “face” as a complicated phenomenon in Confucian societies. Indeed, the feeling of face is highly context dependent. One may have very different (having or losing) face perception if the same face event occurs in a different context. To better capture the features of how face is felt, effects on possible responses need to be considered. Therefore, this article adopts a perspective of psychosocial equilibrium to elaborate people’s feeling of face in Taiwan, a Confucian society. The first section illustrates the concept of psychosocial equilibrium and its psychodynamic effects on people’s feeling of face. Then, the second section of this article takes positive social situations (having face events) as backdrop to exhibit how people balance their psychosocial equilibrium with different relationships. Following the positive social situations, the third section of this article then focuses on the negative situations (losing face events) to explain how losing face is felt due to unbalance of psychosocial equilibrium with one’s relation in that specific context.

“Face” has long been a topic of study in the fields of psychology and sociology (Hu, 1944; Goffman, 1955; Brown, 1968; Ho, 1976; Brown and Levinson, 1987; Chen, 1988; King, 1988; Ting-Toomey, 1994; Zai, 1995). Although Goffman (1955) claimed that “face-work” is one of the universal human needs, comparing to take “face” as politeness and/or freedom of one’s action (Goffman, 1967; Brown and Levinson, 1987), face plays a more complicated role in Confucian societies and is apparently different from that in the West (Ho, 1976; Cheng, 1986; King, 1988; Hwang, 2006; Hwang and Han, 2010). Previous studies on face phenomenon in Confucian societies tend to adopt a more static point of view to describe the concept and contents of face (Ho, 1976; Cheng, 1986; King, 1988; Chu, 1991; Zai, 1995; Kim and Nam, 1998; Hwang and Han, 2010). To name a few, King and Myers (1977) differentiated two types of face: social face versus moral face; He and Zhang (2011), from a different angle, classified *Mianzi* (face) into three different levels: individual, relational, and group. As face has profound implication on people’s daily life, these studies are helpful for an outsider to understand what face is in Confucian societies.

Interestingly, the feeling of face is not a static status; instead, it is a dynamic process and can be affected by different factors in the context it occurs. For example, a student made a presentation for a case study in his/her class but could not properly answer questions proposed by others after his/her presentation. From a static point of view, we know s/he would definitely experience the feeling of losing face. However, if we take a perspective of dynamic process, whether this student would experience the feeling of losing face or how badly s/he would have the sense of losing face would depend on the factors in the context. For example, who proposed those questions, a teacher or a classmate? How was the atmosphere in the classroom, supportive or competitive? Even the way those questions were proposed, friendly or criticizing? All these factors matter because they can all potentially affect how this student interprets his/her situation; and thus, have an impact on this student’s feeling of face. In order to better capture the dynamic features of how face is perceived and works in a Confucian society, this article will elaborate the concept of “psychosocial equilibrium” in the beginning. Then the psychological process for feeling of face, especially from the perspective of how one reacts to maintain his/her psychosocial equilibrium among different relations in a face situation will be illustrated.

## The Concept of Psychosocial Equilibrium

Hsu (1971) proposed a concept of “psychosocial homeostasis” to address man’s relationship with his fellow men in the human mode of existence. To do so, Hsu (1971) divided the psychosociogram of a person into seven irregular, concentric layers, i.e., wider society and culture, operative society and culture, intimate society and culture, expressible conscious, unexpressed conscious, pre-conscious, and unconscious (from Layer 1 to Layer 7, respectively). In these different layers, Hsu named Layers 3 and 4 with slight extension into Layer 2 and 5 “*jen*,” which is the Chinese word meaning “man.” The Chinese conception of man sees the nature of an individual’s external behavior especially in terms of how it fits or fails to fit the interpersonal standards of the society and culture.

“Homeostasis” is a term refers to an individual’s biological tendency to maintain equilibrium across all systems within its body. For example, if a person is too stressed or upset by life’s events, his sympathetic system will be triggered to increase the body’s physiological arousal (into an alert state). However, it is potentially harmful to his body to stay stressed or aroused, the sympathetic and parasympathetic systems will work together to keep the body’s level of arousal in balance for optimum functioning. On the analogy of physical homeostasis, Hsu (1971) emphasized that Layer 3 (intimate society and culture) and Layer 4 (expressible conscious) are the central substance of a person as a social and cultural being. It is the human constant, within which every human individual tends to maintain a satisfactory level of psychic and interpersonal equilibrium, in the same sense that every physical organism tends to maintain a uniform and beneficial physiological stability within and between its parts.

By the same token, Hwang (2011) proposed a model of self named “Mandala.” In this Mandala model, self refers to an individual who has been socialized with the ability of reflexivity; therefore, it is the locus of experience and is able to take various actions in different social contexts. Hwang emphasized that the self exists in a field of forces in one’s life world. When one intends to act, several forces may influence his decision, especially when he identifies with a particular social role. On the one hand, the individual has to think about how to act as a socialized person. On the other hand, he is pushed by various desires for he is also a biological entity. Therefore, when one takes action in a specific situation, he may reflect his disposition to maintain his psychological equilibrium in that specific social context.

Putting Hsu’s (1971) psychosocial homeostasis and Hwang’s (2011) self of Mandala together, it is apparent that they both stress that as social beings, people are simultaneously affected by their inner biological forces and expectations from the outside world in which they live. This article adopts and reconciles the concepts of psychosocial homeostasis and self of Mandala together from Hsu and Hwang and terms it as “psychosocial equilibrium” to elaborate how people react in face situations to balance their inner desires and outward relationships. Han (2010a, 2012a, 2014) conducted a series studies to classify the emotions people experienced in face losing situations and found that although losing face is more a holistic feeling, different emotions may involve depending on the contextual clues in the situations. The different emotions participants experienced (rated) in Han’s studies can be viewed as an empirical evidence to support the existence of psychosocial equilibrium. For example, Han (2014) adopted a scenario experimental method to examine the feeling of face and emotions experienced in an ability failure situation. The ability failure event was kept the same across all episodes; however, the contextual clues for participants to interpret their situations were manipulated. The scenario in this study described a college student “A” who attended a family gathering where all family relatives celebrated a grandparent’s birthday. Bantering together, A’s cousins talked about their schools excitedly. Then Episode one described that A became conscious of inferiority because A was in a not-that-good college while all his/her cousins were in top universities. Episode two was exactly the same except when A became conscious of inferiority, s/he was also aware that his/her mother looked sullen about this. In Episode three, the mother of A compared him/her to his/her cousins for poor academic performance in front of the relatives. Episode four described A’s mother mentioned the same issue to A later that day in A’s bedroom. The results of this study showed that participants rated the highest degree of losing face in the mother’s public comparison; the lowest in self-conscious inferiority. As for the emotions participants experienced, the feelings of depression, embarrassment, and shame were highly rated across all episodes. Feelings of anger and being humiliated appeared mainly in the public comparison context. According to the two-factor theory of emotion, an emotional state may be considered a function of a state of physiological arousal and of a cognitive explanation to this state of arousal. Because the physical states are difficult to label on their own, people will refer to contextual clues to make attributions for the state of arousal (Schachter and Singer, 1962). Following this rationale, the different emotions participants experienced in Han’s (2014) study could be understood as participants experienced a state of physiological arousal due to losing balance of psychosocial equilibrium with their social world; then, they labeled the state of arousal with different cognitive explanations based on the clues in the contexts. To be more specific, psychosocial equilibrium in this article refers to a psycho-status that one has to maintain with his social world. In Confucian societies, the concept of social world mainly refers to different interpersonal relationships one is involved with in daily life. Therefore, when a face event happens, will a person interpret this event as losing or having face depends on whether this person senses losing psychosocial equilibrium with his social world.

Taking psychosocial equilibrium as a framework, the aforementioned a student presenting a case report example will be easy to understand. If a teacher proposed a question and this student could not answer, s/he might feel embarrassed but not necessarily lose face. The reason is students in Confucian societies naturally take their teacher as a superior whom students should respect and be submissive to; therefore, little to no psychological equilibrium would be lost between them. On the contrary, this student should have strong feelings of losing face if a classmate proposed the same question that s/he could not answer. Because a student should psychologically view him/herself as good as his/her peer group, being challenged and/or defeated by a classmate is a threat to the balance of psychosocial equilibrium between this dyad. Losing balance of psychosocial equilibrium is an uncomfortable psychological status that generates psychodynamics and impacts this student’s interpretation of the event and feeling of face. Han (2012a) adopted another scenario experimental method to examine this hypothesis. The scenarios were about a college student who was well prepared (vs. not prepared) for his/her midterm presentation. After the presentation, in the well-prepared episode, a classmate (vs. teacher) asked a very difficult question that s/he could not answer; in the not prepared episode, a basic simple question was asked. The results showed that participants rated the highest level of loss of face in the case where their classmate asked a simple question that they could not answer. On the contrary, the teacher asking a hard question in a well-prepared situation was considered the least harmful to the feeling of face. The results of Han’s (2012a) study supported the perspective that different relations might have very different psychological implications for one’s self-evaluation. An individual might feel losing face in one relation but does not feel the same way in another due to the effects of psychosocial equilibrium among different relationships.

## Psychosocial Equilibrium and Relations

Hwang (1987) proposed a theory model to illustrate Chinese face and favor behaviors. In that model, Hwang emphasized that when a Chinese is interacting with others in a social context, two major things related to role relationships within the interacting dyad should be recognized. The first thing is the superiority of relative status of the dyad; and, the second thing is the degree of closeness between the dyad. Why superiority and closeness are important is because Confucian society is very authority and relation oriented (Yang, 1991; Ho, 1993; Hwang, 2000). People who grew up in this culture naturally internalized cultural values of respecting and submitting to authority (Yang, 1981) and taking the immediate relations around them in the interacting context as a reference framework for behavior guidance (Ho, 1998).

For people’s relationships, Hwang (1987) classified three sorts of interpersonal relationship: instrumental ties, mixed ties, and expressive ties. Expressive ties are generally a relatively permanent and stable social relationship, such as family members; mixed ties include relationships with acquaintances outside the immediate family; and instrumental ties are established mostly by a dyad of strangers for specific purpose(s). Different rules will be applied when one interacts with others. If the interacting other belongs to expressive ties, “need rule” will be applied. According to this rule, every member should do his best for the family, and the family will in turn supply him the resources necessary for living. To be more specific, the rule of need cares more about distributing resources, profits, and/or other benefits to satisfy its members’ legitimate needs, regardless their relative contributions. If the interacting other belongs to instrumental ties, “equity rule” may dominate the interactions. The rule of equity is primarily activated in economically oriented situations and encourages people to allocate resources in proportion to their contributions. In other words, people in this kind of relationship only take it as a means to attain their goals. If the consequences of the social exchange seem unprofitable, one may bargain or even completely break off the relationship without regret. However, if the interacting other is a mixed tie, which means the interacting dyad know each other and may expect to meet the other again in the future (some may be in a daily routine), “*renqing* rule” will be taken into account. The rule of *Renqing* connotes a set of social norms by which one has to abide in order to get along well with other people in Confucian society and it includes two basic kinds of social behavior. The first one is people should keep in contact with the acquaintances in their social network; and the second one is people should sympathize, offer help, and do a “*renqing* (favor)” for a member in their social network if that person gets into trouble or encounters a difficult situation.

**FIGURE 1:**
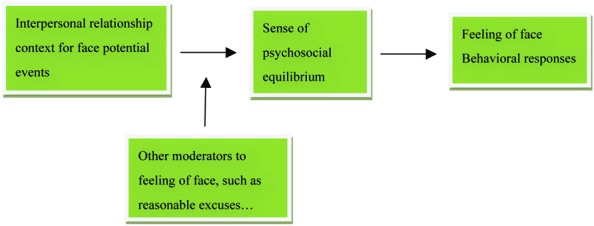
**The role psychosocial equilibrium plays in perception of face**.

On the other hand, instead of classifying different types of interpersonal relationship, Ho et al. (1989) and Ho (1998) claimed that one should adopt “methodological relationalism,” i.e., “person in relations” and “persons in relation” to better understand and interpret Chinese social behaviors. According to Ho et al. (1989) and Ho’s (1998) opinion, one will certainly get involved in a variety of social events. Others involved in those social events constitute his/her “persons in relation.” Then, how s/he perceives these others will form his/her “person in relations.” Hwang’s different relational ties and Ho et al.’s (1989) person in relations can be a contextual framework to understand people’s feeling of face because they both imply the dynamics of psychosocial equilibrium for people in social interaction. As psychosocial equilibrium is a psycho-status that a person has to maintain with his social world, whether a person interprets an event as losing or having face depends on whether this person senses losing equilibrium with his social world. To make it easier to understand, **Figure 1** presents the role psychosocial equilibrium plays in one’s perception of face in his/her social world.

Brown and Levinson (1987) mentioned that “face” is the evaluation of one’s public image after s/he reflects his/her own actions in certain social circumstances. It somehow equals to one’s self-identification in a special situation; therefore, it can be called one’s situated identity (Alexander and Knight, 1971). One may feel losing, maintaining, or increasing face based on recognizing other’s evaluation of his/her behavior in a certain situation. Generally speaking, people’s feeling of face can be basically divided into two domains: having face and losing face. When the face related issue is positive, it is about face having; whereas, if the issue is negative, people will have to struggle to save face. Apparently, positive face issues have very different implications from negative ones for people’s psychological equilibrium.

## Positive Social Situations and Feeling of Face

As cultural psychologists have argued that “interpersonal relationships” and “individuals” are not equally stressed in the East and West (Triandis, 1989; Markus and Kitayama, 1991; Ho, 1998; Crocker and Park, 2004), people who grow up in Western culture are supposed to be more self-affirmative and self-enhancing when sharing their success or achievements with others. On the contrary, people in Confucian societies are supposed to be modest toward their social achievements (Bond et al., 1982; Markus and Kitayama, 1991; Heine et al., 1999, 2000). However, it might be a myth to take it for granted that a modest response will always be applicable and/or suitable for one’s success in Eastern cultures. In Confucian societies, people can be either self-enhancing or modest to a positive event of face because different relations imply different rules for them to follow in order to maintain psychosocial equilibrium with their social world (Hsu, 1971; Hwang, 1987, 2000; Ho, 1998).

While Western Christianity advocates that each person is an independent entity created by God and should therefore strive to defend the territory of self that has been drawn around the immediate surface of the physical body, people in Confucian society tend to view their lives as a continuity of their parents’ lives. As a result, one’s family members, especially parents and children, are more likely to be included in the territories of his/her self. In other words, family members are usually perceived as a single body named “Big-Self” and are especially liable to the feelings of having glory or shame together (Su and Hwang, 2003; Hwang and Han, 2010; Han and Li, 2011). When the face issue is positive, one will be definitely happy to share glory and add face to his/her family because his/her face or honor also belongs to the family, the big self. In this case, it is unlikely that one will be modest or efface his/her success because it would be like s/he is denying the face or honor to the family (Han, 2010b). Therefore, the psychosocial equilibrium here is well maintained when one is sharing face or even bragging the positive issue to enhance the family’s face. Except from family members, one still has to interact with other relations in his/her daily life. Strangers or persons in the relation of instrumental ties are those in which one does not have personal affection involved. Therefore, when the face issue to one is positive, strangers hardly know because there is almost no connection between one and a stranger for his/her personal issue. Even when this positive event is publicized and strangers deliver their admiration or compliments; being polite is enough, no glory sharing or modesty is needed.

Relations with acquaintances or mixed ties are the most complicated relations for one to interact with (Hwang, 1987). Acquaintances can be very different in the degree of closeness; some might be thought of as closer and trustworthy while others might be seen as distant and unfaithful. When the acquaintance is close, the feeling of we-ness between close friends somehow makes the relation like family; and thus, the psychodynamics for the interaction will be simpler. One will be willing to share glory with them and they will be happy for him/her when the face event is positive. Modesty of one’s success in this case is possible but not necessary. If the acquaintance is not that close; then, it will be another story. In that case, one will have to calculate very carefully to figure out the best reaction. Not only because acquaintances are the people with whom there is daily interaction; but also because these acquaintances usually share other social networks. To complicate matters, it is highly possible that face issues will be spreading to those social networks. As a result, face related behavior will not only be judged by acquaintances, but it will also be gossiped about in social networks. Therefore, it would be understandable that one should be modest because others will judge his/her reaction for success by social modesty norms. Self-enhancing responses will make people think of him/her as proud or arrogant.

Following the rationale, it is clear that previous studies suggesting that people in Confucian societies tend to be modest for their social achievement were only partly correct. Han (2010b, 2012b) focused on interpersonal closeness and threat of one’s achievement in interaction with others to examine how these factors affect one’s attribution to his/her achievement. The results of these studies found that one would adopt very different styles of attribution to different relations due to different motivations. When the achievement was a threat to the interacting other, one would attribute his/her achievement to luck, presenting a modest response whether that person is an intimate or not. The concern behind the modest attribution was empathy and saving other’s face as previous references mentioned (Yang, 1981; Pong, 1993; Ho, 1998). It should be noted that when one’s achievement is a threat to others, modesty is a gesture of empathy which is universal, even people in the West will do the same (Tice et al., 1995). The behaviors of empathetic modesty can be addressed from the viewpoint of Hardy and Van Vugt’s (2006) “theory of competitive altruism.” Competitive altruism suggests that people’s attempt to outcompete each other in terms of generosity may be because altruism can enhance the status and reputation of the givers. In deed, the results of Hardy and Van Vugt’s (2006) studies found that the most altruistic members gained the highest status in their group and were most frequently preferred as cooperative interaction partners. Different from the perspective of competitive altruism, this article focuses mainly on the effects of unbalanced psychosocial equilibrium to interpret the behaviors of empathetic modesty. When one’s success or achievement reflects the failure of others (Beach et al., 1998; Tesser, 2000), the psycho status between the interacting dyad is apparently unbalanced. To be modest is a way to rebalance the psychosocial equilibrium with the interacting other, whether the inferior one is intimate or not is not a concern.

If the achievement has no threat to the interacting other; then, one’s attribution to his/her success would depend on the closeness to this interacting person. The findings of Han’s (2010b; 2012b) studies showed that, when the person was an intimate, most participants attributed their success to efforts and ability just as their Western counterparts, presenting a self-enhancing pattern of response. The motivation for this self-enhancing behavior is sharing the glory. In fact, data collected from qualitative interviews in Han’s (2010b) study found that modest responses in this situation were quoted as “not that appropriate” or “somewhat hypocritical” by the interviewees (pp. 14–16). However, when the interacting other was just an acquaintance, participants would be modest and attribute their achievements to luck. The motivation for being modest in this situation is to abide by social modesty norms, which is different from that of empathy because one’s success has no threat to the interacting person in this case.

Being modest for one’s success is prevailing in Confucian societies, even when the achievement is not a threat to others. Some psychologists adopt a viewpoint of impression management to explain this phenomenon, claiming that the purpose of being modest is to earn others’ positive evaluation such as “decent upbringing” (Pong, 1993; Wang and Sun, 2007). On the other hand, some psychologists view modesty as a powerful social norm in East Asia. People are taught not to boast of their abilities or accomplishments in order to maintain interpersonal harmony (Yang, 1981; Ho, 1998; Kurman, 2001, 2003; Cai et al., 2007). Both views of impression management and modest social norm fit the perspective of psychosocial equilibrium. The intriguing part here is that modesty can also play a role to enhance one’s self-worth and feeling of face through the responses of interacting others. For example, Muramoto (2003) found an “indirect self-enhancement” among Japanese that although participants in Japan attributed their success to external factors (luck, chance, and environment), showing a tendency of self-effacing, they would expect their intimates such as close friends and family members to emphasize the internal factors (ability, effort, and personality) to support their positive self-regards. On the contrary, those non-significant others were not expected to protect or enhance the self-worth of the achiever.

However, Han (2011) found that, in Taiwan, the face or self-esteem one lost from being modest could be well compensated through the latter part of social script executed by the interacting other (the admirer). In order to exhibit the dynamic face having process in social script, Han (2011) instructed her participants to recall an episode in which they had a success or accomplishment and their friend (just an acquaintance, not intimate) complimented them. If the participants had not had this kind of experience, they were instructed to imagine how people in such a situation might respond. The participants were then instructed to write down the conversations between the interacting dyad where the achiever was the participant and the admirer was their friend. The results of this study found that although some participants used “I am not that good” or “nothing special” to deny their accomplishments, most participants attributed their success to external factors such as luck, others’ help, or team work. Apparently, the achievers were taking a social modesty norm as their behavioral standard. However, if the latter part of this social interaction was examined, we would also find that most of the admirers’ responses to the achievers were to challenge the achievers’ modesty by using phrases such as “you are much better than the opponents” or “your performance was perfect” to express their compliments more intensively. In other words, the modest achiever can enhance his/her feeling of face through the admirer’s repeated compliments and maintain interpersonal harmony as well.

The reason why the modest person can both enhance his/her face and still maintain interpersonal harmony is also due to the function of psychosocial equilibrium. It is important to understand that the psychological process in a social interaction is always dynamic. If one encounters an acquaintance to admire his/her success or achievement and does not behave modestly, the psychosocial equilibrium between the interacting dyad is hurt. By the same token, when the achiever initially plays a modest role in a social script as being modest, the role for the admirer to take in the latter part of the social script is to re-enhance the face in front of the achiever by expressing the compliments more intensively. Failing to do so will also jeopardize the psychological balance between the interacting dyad.

## Negative Social Situations and Feeling of Face

Comparing positive events such as achievement can add glory to one’s face, negative issues threaten one’s face and make the situation awkward; therefore, they are more stressful and complicated in reactions. It is obvious that one will avoid or try to alleviate the harm from losing face if s/he has a choice. Under the concept of Big Self, if the face event one encounters is negative, s/he will not only harm his/her own face, but also put his/her family’s face under threat. It is very possible that this person will cover this event from his/her family members because when one hurts the face of his/her family, s/he will be in a state of guilt for being the black sheep in the family. In addition, even though family members are supposed to give supportiveness when one is in need, it still embarrasses this person and the guilt feeling will be reminded every time s/he faces his/her family members if they are aware of the face losing event s/he has done.

Except from family members, one still has to interact with other relations in his/her daily life. It is natural for one to hide a negative event even from strangers to protect his/her feeling of face. However, if loss of face is inevitable, one has to find others to help solve that face threatening issue. Strangers or persons of instrumental ties will be one of the best choices. Again, the reason is psychosocial equilibrium that one has to maintain psychological balance with his/her social world. Persons of instrumental ties do not know who you really are, and actually, you are just one of their clients who all have similar troubles. In addition, the relation can be terminated as soon as the trouble has been solved. After that, one can again function in his/her life world as if nothing had ever happened.

When a face losing situation is foreseen but still inevitable, losing face in front of the most harmless person would be a plausible strategy for one to adopt. However, if one does not want or trust help from people of instrumental ties, close friends will be an alternative choice. One thing should be noted in this case is that, close friends are not family members; therefore, they will not suffer from the loss of face. In addition, close friends will cover the event for us because the code of brotherhood demands that we should help and keep secrets for our friends (Yang, 1991). After being helped by close friends, although one will feel indebted which make his/her psychosocial equilibrium unbalanced, the rule of *renqing* (favor) will work to recover the psychological balance between the dyad in the long run (Hwang, 1987).

As one’s interaction with others of instrumental ties can and will terminate at the end of that event; interaction with others of expressive ties might be stable and durable (Hwang, 1987). Family is the most important relation of expressive ties for people in Confucian societies. Maintaining psychosocial equilibrium with family members plays an important part in people’s daily lives. It will be reasonable to hypothesize that comparing relations of mixed and instrumental ties, when a negative event happens which may hurt a person’s face, his/her family member will be the one they want to hide that event from most. To examine this hypothesis, two scenarios were constructed in one study of Han and Li’s (2008) research. In one scenario, they described a person in the story who found that s/he was infected with venereal disease; while the same person had gallstones in the other scenario. The participants were instructed to imagine that they were the person in the scenario and had to choose a medical doctor from different relations (i.e., older brother/sister, classmate in senior high, and/or the doctor who is a stranger) for their disease. The results of this study showed that participants in different scenarios made very different choices of doctor. To be more specific, when the disease was sexually contracted which might be a threat to one’s face, a “from far to near” helper seeking pattern was exhibited; most of the participants (91%) chose the stranger doctor to help. The remaining 9% chose a friend of a mixed tie, and no one chose a family member. The reason for participants looking for help from strangers was because they intended to “save their face,” hoping that the loss of face from their contracted disease would not be exposed to the stable and durable relations such as family members and/or acquaintances in order to maintain the psychosocial equilibrium among them. Interestingly, getting better help is relatively not a concern in this situation. It was another story when the disease involved was gallstones, which has nothing to do with one’s feeling of face. In that case, most participants (92%) chose a helper from family members of an expressive tie and no one chose a stranger. In this situation, participants based their choice of helper on the importance of “better help” and presented a “from close to distant” order of seeking help. The results of Han and Li’s (2008) study implied that the need for people to maintain psychosocial equilibrium among different relations is not the same. Family members and friends are those one has to face all the time; therefore, when an issue might put his/her face under threat, strangers spontaneously become a better choice, for it can be terminated as soon as the issue ends.

There are two types of face in Confucian societies: social face and moral face (Cheng, 1986). “Social face” is gained through the status achieved by one’s talent, endeavors, and/or ability. “Moral face” refers to the social evaluation of one’s moral character, which is the baseline of one’s integrity of personality (Hwang, 2006). One may argue that the reason why venereal disease has such dramatic effects on people’s helper choice is because it is related to sexual morality that is the baseline for one to function in his/her community (Su and Hwang, 2003; Hwang and Han, 2010). Negative events related to social face would not have similar effects on people’s decision making because the social standard for ability performance is more lenient and flexible. To answer this question, Han and Li (2008) constructed another two scenarios of social face event to examine whether participants would still exhibit the pattern of “from far to near” for seeking a helper. One non-threatening social face scenario described a father who was not capable of paying his two children’s college tuition because he was robbed on the way to the bank; the second scenario was considered a threat to social face as it described a father who was not able to afford children’s tuition because he could not save enough money. Participants had to choose a helper from different relations (i.e., family members, friends/colleagues, and/or banks). The results of this study were similar to the results of moral face (Venereal disease vs. gallstones) though the effect was not that overt.

Although moral face is more important than social face in terms of losing face, one may choose not to strive for social face, but must protect moral face in all situations (Hwang, 2006). The similar results in participants’ choice of helper for both moral and social face situations in Han and Li’s (2008) research highlighted the fact that, in Confucian societies, one’s immediate responses and feeling of face to a specific social event are affected by the psychosocial equilibrium s/he perceived immediately in that context, not the type of face. In other words, to differentiate the importance from moral face to social face is more like static knowledge stored in one’s mind; however, when one really encounters a situation which threatens his/her feeling of face, it would be an automatic response to the factor(s) that jeopardize his/her psychosocial equilibrium with his/her immediate social world. That impacting factor(s) could be either moral face or social face, or both.

## Moderators to Feeling of Face

Although losing face is harmful to one’s self-image and dignity, it is very sensitive to contextual clues. Some factors other than interpersonal relationships in a face threatened situation might worsen or alleviate the face losing feeling. For example, the lack of personal closeness will moderate one’s feeling of face when s/he is in a face losing situation. Liu (2007) found that if one has to give negative feedback like poor academic performance to a friend, expressing the message privately would be better than delivering the same message in front of others. Because delivering negative feedback publicly implies one’s weak points will be revealed in front of others, which will definitely harm one’s feeling of face. Corresponding to Liu’s (2007) study, Han (2014) also found that, compared with being blamed privately, participants in her study rated stronger feelings of losing face if their mother condemned them for poor academic performance in front of family relatives.

On the other hand, whether one has a reasonable excuse for why s/he is distressed for losing face can also diminish the threat to the face. As Han and Li’s (2008) study showed, although not capable of paying children’s tuitions was stressful for a father, participants in the being robbed scenario did not have the feeling of losing face. On the contrary, not being capable of saving enough money for children’s tuitions was rated by participants as highly harmful to one’s feeling of face. The results of this study implied that being robbed is a reasonable excuse, which could save one from the harm of losing face. Actually, finding reasonable excuses or preferring private occasions for negative messages in order to protect one’s self-image are universal; even people in the West will have similar responses. For example, Brown and Garland (1971) manipulated an embarrassing situation in which participants would be observed either by a stranger or by a friend. The results of this study showed that, compared with the situation of being observed by a stranger, participants were more willing to give up their money rewards to prevent their friend from observing them in an awkward situation. The reason why participants in Brown and Garland’s (1971) study were more concerned about their self-image in front of their friends could be understood by the concept of psychosocial equilibrium. Interaction with strangers could be terminated immediately after the experiment; however, relationships between friends would not finish with the experiment and would make the feeling of embarrassment last much longer.

On the other hand, because authority-orientation is a feature of Eastern culture, it may have an effect on people’s feeling of face. Hofstede (1980) proposed four dimensions of work-related values on which the differences among national cultures can easily be understood. These four dimensions of values are: “power distance,” “uncertainty avoidance,” “individualism/collectivism,” and “masculinity/femininity.” Nations in Confucian societies such as Taiwan are relatively high in power distance and low in individualism. Yang (1991) proposed that the reason why Chinese people are sensitive to the existence of authority is due to the patriarchal system in the society. As a matter of course, people are sensitive to the existence of authority and spontaneously obey the orders from superiors (Han et al., 2005; Wu et al., 2008; Chien, 2013).

Following this rationale, it would be easy to imagine that, if a face offensive situation is caused by a superior, the feeling of losing face or being humiliated would be less than that caused by a peer. The results of Han’s (2012a) study supported this hypothesis. In this study, participants were instructed to imagine that they were blamed for not being well prepared for a term presentation by either a teacher or a classmate to induce the feeling of losing face. Indeed, the results of this study showed that, the feeling of losing face was significantly higher when the blaming was from a peer than from a teacher. Again, the face losing effect in Han’s study could be explained by the concept of psychosocial equilibrium. Comparing with being blamed by a peer, being corrected or scolded by a superior is usually taken for granted by people in Confucian societies; therefore, it would be less likely to harm one’s psychological balance with the superior. In another study, although not directly measuring feelings of face, Han et al. (2005) recruited graduate students in Taiwan as participants. In that study, participants had to decide whether they would accept or reject an unreasonable favor-doing request from a professor (vs. a classmate); then, they had to rate their feeling of being offended. The results showed that participants were more likely to accept an unreasonable request of favor from a professor than a classmate. In addition, they rated lower feelings of being offended when the unreasonable request of favor was from a professor.

## Conclusion

This article adopted a perspective of psychosocial equilibrium and systematically focused on different relations to elaborate people’s feeling of face and possible responses in face situations. However, as face is a prevailing and complicated phenomenon in Confucian societies, varied factors affect people’s feeling of face and their range of responses. Face phenomena can be understood from different ways. For example, some researches tried to illustrate people’s face behaviors from a viewpoint of personality character of face orientation, such as “thin-skinned” vs. “thick-skinned.” In general, one who is “thin-skinned” is more likely to feel “losing face” when his/her misconduct is exposed. On the other hand, one who is “thick-skinned” is less likely to have such a feeling (Chen, 1988; Hwang and Han, 2010). Chou (1996) also classified the face concern into two major face orientation types: protective and acquisitive. Furthermore, Chou also developed a Protective and Acquisitive Face Orientation Scale to examine people’s face orientation in Confucian societies. There are still some studies focused on the connection of feeling of face and related social behaviors, such as gift-giving (Bao et al., 2003; Qian et al., 2007) and conspicuous consumption (Bao et al., 2003; Li and Su, 2007; Zhang et al., 2011) in China. All of these studies have offered different angles to understand the face phenomenon and how people perceive their feeling of face in Confucian societies.

It should be noted that, face events and their effects on people’s psychosocial equilibrium in this article are limited to the person him/herself. Similar face events only involving others, such as family members, close friends, or classmates (Bedford and Hwang, 2003; Su and Hwang, 2003; Chu, 2008; Han and Li, 2011) and their possible effects on people’s “face of Big Self” were not discussed in this article.

## Author Contributions

The author confirms being the sole contributor of this work and approved it for publication.

## Conflict of Interest Statement

The author declares that the research was conducted in the absence of any commercial or financial relationships that could be construed as a potential conflict of interest.
